# Acoustic Wave Sensor Detection of an Ovarian Cancer Biomarker with Antifouling Surface Chemistry

**DOI:** 10.3390/s24247884

**Published:** 2024-12-10

**Authors:** Katharina Davoudian, Sandro Spagnolo, Edmund Chan, Tibor Hianik, Michael Thompson

**Affiliations:** 1 Department of Chemistry, University of Toronto, 80 St. George Street, Toronto, ON M5S 3H6, Canada; k.davoudian@mail.utoronto.ca (K.D.); edmund.chan@mail.utoronto.ca (E.C.); 2 Faculty of Mathematics, Physics and Informatics, Comenius University, Mlynská dolina F1, 842 48 Bratislava, Slovakia; spagnolo2@uniba.sk (S.S.); tibor.hianik@fmph.uniba.sk (T.H.)

**Keywords:** lysophosphatidic acid, gelsolin, actin, ovarian cancer, antifouling linker, thickness shear mode, biosensor

## Abstract

Ovarian cancer (OC) must be detected in its early stages when the mortality rate is the lowest to provide patients with the best chance of survival. Lysophosphatidic acid (LPA) is a critical OC biomarker since its levels are elevated across all stages and increase with disease progression. This paper presents an LPA assay based on a thickness shear mode acoustic sensor with dissipation monitoring that involves a new thiol molecule 3-(2-mercaptoethanoxy)propanoic acid (HS-MEG-COOH). HS-MEG-COOH is an antifouling linker that provides (a) antifouling properties for gold substrates and (b) linking ability via its terminal carboxylic acid functional group. The antifouling ability of HS-MEG-COOH was tested in whole human serum. The new molecule was applied to the LPA assay in conjunction with a spacer molecule, 2-(2-mercaptoethoxy)ethan-1-ol (HS-MEG-OH), in a 1:1 *v*/*v* ratio. HS-MEG-COOH was covalently linked to gelsolin–actin, a protein complex probe that dissociates due to LPA-binding. LPA was detected in phosphate-buffered saline and undiluted human serum and achieved a low limit of detection (1.0 and 0.7 μM, respectively) which was below the concentration of LPA in healthy individuals. The antifouling properties of HS-MEG-COOH and the detection of LPA demonstrate the ability of the sensor to successfully identify the early-stage OC biomarker in undiluted human serum.

## 1. Introduction

Ovarian cancer (OC) is commonly diagnosed in advanced stages since available diagnostic methods are unreliable for early-stage detection. A screening test is critical for identifying the disease when the 5-year survival rate is ~92% for localized OC, rather than ~31% for metastasized OC [1]. Currently, the only clinically available blood test measures cancer antigen 125 (CA-125), which is upregulated in merely 50% of early-stage patients and therefore not useful for early detection, even when combined with transvaginal ultrasound scanning (TVS) [2,3]. In fact, annual TVS and CA-125 testing have been shown to have no impact on decreasing OC mortality rates [2,3]. When combined, TVS and CA-125 testing provide positive predictive values ranging from 35 to 43%, depending on the type of ovarian cancer [4]. Since CA-125 blood tests are not suitable for identifying early OC, detecting this disease depends on a physical exam, biopsies, or imaging techniques such as ultrasonography, magnetic resonance imaging, and computerized tomography scanning [2,5,6]. The biomarker lysophosphatidic acid (LPA) presents an opportunity for the diagnosis and prognosis of OC, since LPA is upregulated and increases in concentration with OC progression [7].

Our developed LPA assay uses an acoustic detection method: thickness shear mode with dissipation monitoring (TSM-D). The sensing area of TSM-D is the surface of a piezoelectric quartz crystal that has gold electrodes deposited on each face. TSM-D measures changes in resonance frequency that can be correlated with mass according to the Sauerbrey equation, which is applicable for rigid or minimally viscoelastic films [8]. For example, if mass adsorbs on the surface of the crystal, it is detected by a decrease in resonating frequency. The sensor also detects variations in dissipation, which enables the analysis of the viscoelasticity of the immobilized adlayer on the crystal surface. More flexible layers have greater deformation, causing an increase in energy loss from the surface, therefore increasing the change in dissipation [8,9]. Together, frequency and dissipation shifts measured by TSM-D can provide insight into the surface interactions of the crystal, which can be applied as an acoustic biosensor.

The major advantages of acoustic biosensors include high sensitivity and specificity, label-free detection, real-time monitoring of mass changes on the sensing surface, and cost-effectiveness [10]. Low levels of analytes can be detected with high sensitivity through minute changes in mass or viscoelasticity on the surface, while the surface can be functionalized with probes that have high specificity for a given analyte. As a result, label-based sensing is not required since real-time mass sensitive monitoring is combined with high-affinity interactions between the probe and the target, reducing cost. Cost-effectiveness is also achieved through the low quantities of materials that are required to functionalize the sensing surface [11].

**Figure 1:**
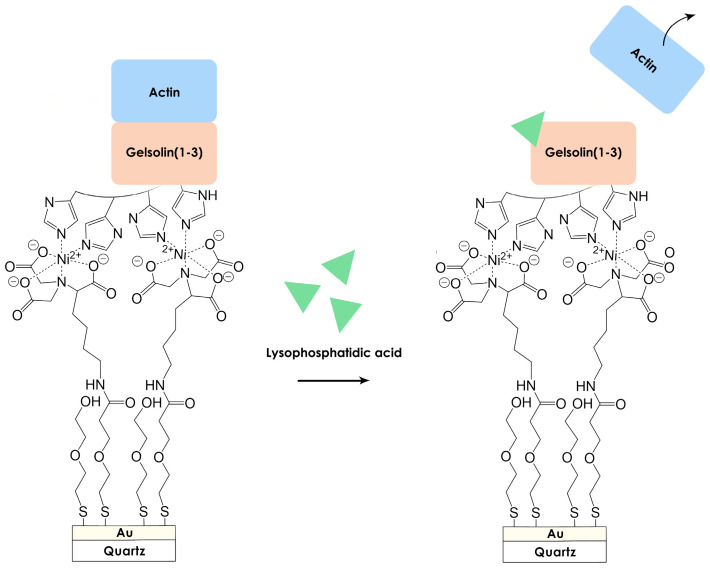
A representation of the biosensor surface and the detection of LPA through the removal of actin protein.

A major challenge for mass-sensitive acoustic biosensors is their low sensitivity for detecting small molecules like LPA [12]. However, LPA’s relationship with a large protein complex, gelsolin(1-3)–actin (with a molecular weight ~81 kDa [13,14]), enables the indirect quantitative detection of LPA. Gelsolin(1-3) consists of the first three domains of gelsolin, a six-domain protein that functions to regulate the polymerization and depolymerization of actin. When LPA binds to gelsolin(1-3)–actin, it separates the complex using electrostatic and hydrophobic interactions [15], causing the loss of actin mass, which increases the resonating frequency of the TSM-D crystal. Gelsolin(1-3)–actin was applied as an LPA probe in various biosensors—an electromagnetic piezoelectric acoustic sensor (EMPAS), chemiluminescence-based iron oxide nanoparticles [16], and fluorescence-based silica nanoparticles [17]. Like EMPAS crystals, gelsolin(1-3)–actin is anchored to the surface of TSM-D crystals via linking molecules (Figure 1). However, unlike EMPAS quartz discs, gold-coated TSM-D discs require thiol linkers for surface modification.

**Figure 2:**
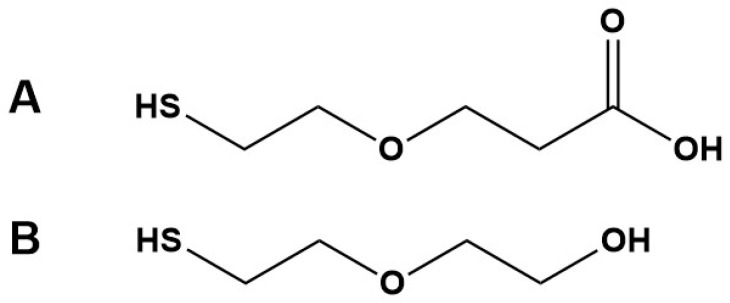
(**A**) The antifouling thiol linker 3-(2-mercaptoethanoxy)propanoic acid (HS-MEG-COOH) and (**B**) the antifouling thiol spacer 2-(2-mercaptoethoxy)-ethanol (HS-MEG-OH).

Thiol surface chemistry is a common method used for anchoring a probe like gelsolin(1-3)–actin to the gold surface of TSM-D crystals. In this work, a new thiol antifouling linker was synthesized, characterized, and applied for LPA detection. Figure 2A illustrates the new molecule, 3-(2-mercaptoethanoxy)propanoic acid (HS-MEG-COOH), which has both linking and antifouling properties, facilitating (1) the immobilization of probes through its terminal carboxylic acid moiety and (2) biosensing in complex, untreated biological samples like saliva or serum. Antifouling is crucial for biosensors to overcome the common challenge of the non-specific adsorption (NSA) of unwanted species, such as the fouling of proteins in milk on the sensing surface. NSA detrimentally affects the performance of a biosensor, which includes impacting its reproducibility, specificity, and sensitivity [18]. Binding antifouling molecules to sensing surfaces can significantly reduce NSA due to the formation of a water barrier—a hydration zone of water molecules that develop on an antifouling surface—that makes unwanted adsorption thermodynamically unfavourable [19,20]. An example of an antifouling thiol linker is 3-dithiothreitol propanoic acid (DTT_COOH_), a branched thiol molecule that may facilitate hydration by the spaces between adjacent DTT_COOH_ molecules, allowing water molecules to become inserted and interact with the polar groups of DTT_COOH_ [21]. Antifouling layers enable biosensors to detect target molecules in complex mediums, making conventional time-consuming sample preparation unnecessary, therefore facilitating rapid, sensitive, and cost-effective detection. While some acoustic biosensors have been developed for detecting OC biomarkers, they are still limited by complex antifouling strategies, which reduce sensitivity and require more time-consuming sample preparation [22,23].

In this study, a mixture of antifouling thiol molecules was applied to build a biosensor for directly measuring LPA in unprocessed human serum samples. The mixture involved HS-MEG-COOH and a previously designed and characterized thiol, 2-(2-mercaptoethoxy)-ethanol (HS-MEG-OH) [24], illustrated in Figure 2B. HS-MEG-OH has antifouling properties but cannot behave as a linker due to its terminal hydroxyl group. Therefore, the thiol mixture provides antifouling spacer molecules (HS-MEG-OH) and antifouling linker molecules (HS-MEG-COOH) to anchor the gelsolin(1-3)–actin protein probe for identifying LPA.

## 2. Materials and Methods

### 2.1. Materials

Unless otherwise specified, all materials were purchased from Sigma-Aldrich (Oakville, ON, Canada). The expression of recombinant gelsolin(1-3) was conducted as previously reported [17], which produces a solution of gelsolin(1-3) in HEPES buffer (20 mM HEPES and 1 mM CaCl_2_ at pH 7.4). Lyophilised actin was solubilized in Milli-Q water (resistivity: 18.20 MΩ.cm). Lysophosphatidic acid (LPA) was dissolved in a solution of 19:1:1 *v*/*v* of chloroform–methanol–acetic acid, separated into aliquots, evaporated, sealed in an inert environment, and then stored at −18 °C. Prior to measuring LPA, 1.5 mL of phosphate-buffered saline (PBS) or undiluted human serum was used to resuspend one aliquot. A healthy male donor voluntarily provided blood for this work’s measurements. Genetic or metabolic information from the blood sample was not collected. This study was conducted in accordance with the ethical principles set forth in the Declaration of Helsinki, and the study protocol was approved by the Institutional Ethics Committee at the St. Elizabeth Cancer Institute in Bratislava (Approval No. 03-2020/EK OÚSA, signed on 4 March 2020).

The synthesis of HS-MEG-OH followed previously published methods [24]. Anhydrous magnesium sulfate, sodium thiosulfate, sodium carbonate, sodium iodide, sodium chloride, and absolute ethanol were supplied by the University of Toronto (Toronto, ON, Canada). All other organic solvents (methanol, ethyl acetate, hexane, dichloromethane, and acetonitrile) were analytical-grade and purchased from Sigma-Aldrich (Darmstadt, Germany), as well as T-butyl acrylate, hydrogen peroxide, zinc, trifluoroacetic acid, β-mercaptoethanol, N-hydroxysuccinimide (NHS), 1-(3-(dimethylamino)propyl)-3-ethylcarbodiimide hydrochloride (EDC), and ethanolamine, which were used without further purification. Ammonium hydroxide and N-Benzyltrimethylammonium hydroxide (40% in methanol *w*/*w*) were purchased from Fisher Scientific (Ottawa, ON, Canada). All aqueous solutions were prepared using Milli-Q water. Phosphate-buffered saline (PBS) was used as a binding buffer and was prepared with 137 mM NaCl, 2.7 mM KCl, 10 mM Na_2_HPO_4_, and 1.8 mM KH_2_PO_4_ and adjusted to pH 7.4. All solutions were filtered with a 0.22 µm membrane (Merck-Millipore, Darmstadt, Germany).

### 2.2. Synthesis of 3-(2-Mercaptoethanoxy)propanoic Acid (HS-MEG-COOH)

#### 2.2.1. Synthesis of 2,2′-Disulfanediylbis(ethan-1-ol) (DS-2EtOH)

**Scheme 1:**
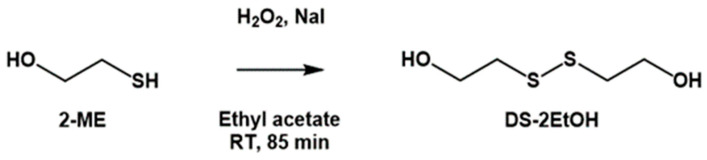
Oxidation of 2-mercaptoethanol.

Hydrogen peroxide (30% *w*/*w* in water, 6.34 mL, 7.04 g, 62.1 mmol) was added to a stirring solution of 2-mercaptoethanol (2-ME) (99%, 4.42 g, 56.0 mmol) and sodium iodide (42 mg, 0.282 mmol) in ethyl acetate (50 mL). The reaction mixture was left to stir for 85 min at room temperature, during which the solution changed from colourless to orange-brown. The solution was then quenched with saturated aqueous sodium thiosulfate (3 × 2 mL) until the solution turned colourless. The organic phase was subsequently washed with saturated aqueous sodium carbonate (32 mL) and saturated aqueous sodium chloride (32 mL). After further drying with anhydrous magnesium sulfate and subsequent filtration, the ethyl acetate layer was concentrated under reduced pressure, yielding DS-2EtOH as a clear colourless oil (3.65 g, 84% yield) (Scheme 1). ^1^H NMR and ^13^C NMR spectra confirmed the success of the reaction, as well as the purification of the product (Figures S1.1 and S1.2 of the Supplementary Materials). The data were consistent with those reported in the literature [25].

#### 2.2.2. Synthesis of Di-tert-butyl-3,3′-((Disulfanediylbis(ethane-2,1-diyl))bis(oxy)) Dipropionate (DS-2EtOEtCOTBu)

**Scheme 2:**
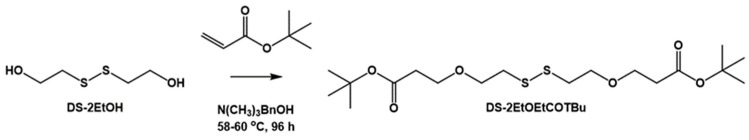
Conversion of oxidized 2-mercaptoethanol to DS-2EtOEtCOTBu.

In an oven-dried flask, N-Benzyltrimethylammonium hydroxide (40% *w*/*w* in methanol, 0.7 mL, 0.644 g, 1.54 mmol) was concentrated under vacuum to dryness and then cooled to room temperature. DS-2EtOH (1.403 g, 9.10 mmol) was added to the resulting residue. The reaction mixture was stirred at room temperature for 15–30 min under an inert atmosphere. Tert-butyl acrylate (1.97 mL, 1.72 g, 13.43 mmol) was added to this mixture. The reaction mixture was heated to 58–60 °C with stirring for 24 h, after which additional tert-butyl acrylate (1.51 mL, 1.32 g, 10.31 mmol) was added. The mixture was held at 58–60 °C for an additional 72 h (total of 96 h reaction time) before finally being filtered through a silica plug. A further purification of the filtered residue by flash silica column chromatography (1:1 *v*/*v* hexane–ethyl acetate) finally yielded the compound DS-2EtOEtCOTBu (0.68 g, 18.2% yield) (Scheme 2). ^1^H NMR and ^13^C NMR spectra confirmed the success of the reaction, as well as the purification of the product (Figures S1.3 and S1.4 of the Supplementary Materials).

#### 2.2.3. Synthesis of 3,3′-((Disulfanediylbis(ethane-2,1-diyl))bis(oxy))dipropionic Acid (DS-2EtOEtCOOH)

**Scheme 3:**
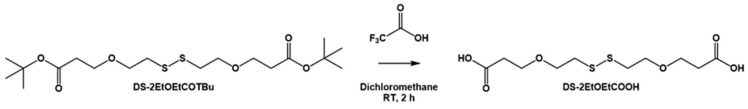
The deprotection reaction step for producing DS-2EtOEtCOOH.

DS-2EtOEtCOTBu (0.502 g, 1.22 mmol) was dissolved in dichloromethane (8 mL), and six equivalents of trifluoroacetic acid (0.560 mL, 0.834 g, 7.32 mmol) was added dropwise to the mixture. The reaction solution was stirred at room temperature. After one hour, three additional equivalents of trifluoroacetic acid (0.280 mL, 0.417 g, 3.66 mmol) was added to the reaction mixture, and the mixture was stirred for an additional hour. The volatiles were evaporated under reduced pressure, and the residue was repeatedly diluted with more dichloromethane and concentrated under reduced pressure to completely remove residual trifluoroacetic acid from the product. The reaction yielded DS-2EtOEtCOOH (0.2564 g, 70.4% yield) (Scheme 3). ^1^H and ^13^C NMR spectra confirmed the purification of the final product (Figures S1.5 and S1.6 of the Supplementary Materials).

#### 2.2.4. Synthesis of 3-(2-mercaptoethanoxy)propanoic Acid (HS-MEG-COOH)

**Scheme 4:**
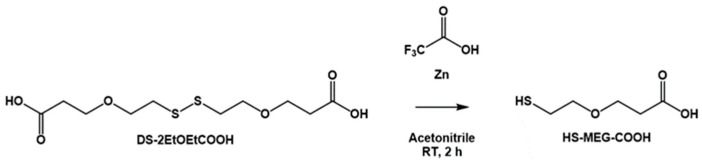
The reduction reaction step for producing HS-MEG-COOH.

DS-2EtOEtCOOH (450.0 mg, 1.5 mmol) was added to a stirred solution of 1% trifluoroacetic acid in acetonitrile (100 mL). Subsequently, powdered zinc (800 mg, 12 mmol) was added to the solution. This mixture was stirred for 2 h at room temperature. The solution was then centrifuged at 7500 RPM for 10 min. The supernatant was decanted and then concentrated under reduced pressure. The residue was repeatedly diluted with dichloromethane and concentrated, finally yielding HS-MEG-COOH (351.0 mg, 77.9% yield) (Scheme 4). ^1^H and ^13^C NMR spectra confirmed the purification of the final product (Figures S1.7 and S1.8 of the Supplementary Materials).

### 2.3. FTIR Analysis of HS-MEG-COOH

Fourier-transform infrared spectroscopy (FTIR) was performed to analyze the linker molecule HS-MEG-COOH. Measurement was carried out using a Prestige-21 FTIR spectrometer (Shimadzu, Kyoto, Japan) in transmittance mode using a CaF_2_ cell (Specac, Orpington, UK). The sample was prepared by solubilizing the linker in ethanol at a concentration of 4 mM. Drops of the sample were deposited on the cell, and the measurement was conducted after solvent evaporation.

### 2.4. Cleaning of TSM-D Crystals

TSM-D crystals (AT-cut with gold electrodes, a sensing area of 0.2 cm^2^, and a fundamental frequency of 8 MHz) were purchased from Total Frequency Control Ltd. (Storrington, UK) and cleaned following previously published methods [21]. A 70 °C basic piranha solution (1:1:5 *v*/*v* of 28–30% NH_4_OH:30% H_2_O_2_:Milli-Q water) was used to clean each crystal in three 25 min cycles. After each cycle, Milli-Q water was used for washes. Two Milli-Q water washes and two methanol washes followed the final basic piranha cleaning.

### 2.5. Functionalization of TSM-D Crystals

The surfaces of the clean TSM-D crystals were individually functionalized with 2 mL 500 µM solutions of HS-MEG-OH, HS-MEG-COOH, or a mix of the two thiols (labelled HS-MEG-Mix, 500 µM each) in absolute ethanol overnight. A 1:1 *v*/*v* ratio of HS-MEG-COOH:HS-MEG-OH was chosen due to its optimal antifouling properties (Section S3 in the Supplementary Materials). The following day, the crystals were incubated with 20 mM N-hydroxysuccinimide (NHS) and 50 mM 1-(3-(dimethylamino)propyl)-3-ethylcarbodiimide hydrochloride (EDC) in water for 35 min. After two Milli-Q rinses, the crystals were functionalized with 2 mg/mL N(α), N(α) bis-(carboxymethyl)-L-lysine disodium salt monohydrate (ab-NTA), and 2 mg/mL NiCl_2_ (Ni-NTA) in PBS for one hour. The crystals were then rinsed twice with Milli-Q water and dried under a gentle flow of nitrogen.

### 2.6. Contact Angle Goniometry

A total of 7 μL of Milli-Q water droplets was used in ambient conditions to measure the contact angle of bare and functionalized TSM-D discs. Contact angle goniometry was conducted with KSV CAM 101 (KSV Instruments Ltd., Helsinki, Finland). All measurements were repeated at least three times and the results were averaged.

### 2.7. TSM-D Set-Up, Measurements, and Data Analysis

The set-up for TSM-D involves an acrylic flow-through cell (JKU, Linz, Austria) in which the functionalized crystal is inserted. The cell is tightened with a holder that connects the two electrodes of the crystal to a vector analyzer SARK-110 (Seeed, Shenzhen, China). When connected to a computer, the analyzer allows for the excitation of the quartz crystal at 8 MHz and the collection of data with Python software (version 3.6.7) [26].

Measurements were made in PBS buffer which was pumped through the inner chamber with a syringe pulled by a Genie Plus pump (Kent Scientific, Torrington, CT, USA) with a constant flow of 50 μL/min. Human serum was used for the antifouling experiments. The holder enables the crystal to be electrically connected to an analyzer and a computer, allowing the resonance frequency to be monitored. The PBS buffer flowed until the resonance frequency reached a stable baseline.

Antifouling experiments were conducted against various surfaces: HS-MEG-COOH, HS-MEG-OH, a mix of these two thiols (1:1 *v*/*v*, labelled HS-MEG-Mix), and crystals with the gelsolin(1-3)–actin sensor surface. Once a stable baseline was reached, the running buffer was replaced with 250 μL of human serum for five minutes. Afterwards, PBS buffer was injected to rinse the crystal surface.

For LPA detection experiments, crystals coated with Nickel–NTA were functionalized in flow with gelsolin(1-3)–actin for 60 min at 20 μL/min. PBS buffer was then flowed to rinse the surface and achieve a stable baseline before exposing the crystal surface to LPA solutions in PBS and human serum spiked with LPA (0.5, 1, 5, 10, 25, and 50 μM) for five minutes. After the incubation of the LPA spiked samples, PBS buffer was flowed until a stable baseline was achieved. Each experiment was conducted in triplicate. The data obtained from the TSM-D instrument were analyzed using a Python code based on the equation of Yoon et al. [27] using Excel (Microsoft^®^), while data analysis and statistical processing were performed using OriginPro 8 (OriginLab Corporation, Northampton, MA, USA).

## 3. Results and Discussion

### 3.1. FTIR Results of HS-MEG-COOH

The structure of the synthesized molecule representing HS-MEG-COOH was analyzed by FTIR to confirm the presence of its functional groups (see Supplementary Materials for details, Section S2). The infrared spectroscopy analysis clearly highlighted the presence of the functional groups in the molecule (Figure S2.1).

### 3.2. Surface Characterization of Functionalized TSM-D Crystals with Contact Angle Goniometry

Following the surface modification of previously cleaned TSM crystals, it was necessary to confirm the formation of the self-assembled monolayer using contact angle goniometry. Fouling tests on bare surfaces or surfaces coated with aliphatic thiols were already performed in a previous study [21]. Therefore, we focused on the analysis of new layers: HS-MEG-COOH, HS-MEG-OH, the thiol mixture HS-MEG-Mix, and the protein sensor surface.

Based on these analyses (Table 1), the crystals coated with HS-MEG-OH had an average contact angle value of about 31.3° ± 4.8°, while those coated with HS-MEG-COOH had a larger value, 43.0° ± 2.8°. This slight difference may be due to the bulkier carboxyl group in HS-MEG-COOH, therefore its SAM could be less dense than the HS-MEG-OH layers. In comparison, the mixed SAM with these two thiols showed an average angle that had an intermediate value between the previous ones, 36.8° ± 4.8°. This value was possible to predict and confirms the heterogeneous functionalization of this layer.

HS-MEG-OH shows the highest hydrophilicity compared to HS-MEG-COOH and the thiol mix, likely because HS-MEG-OH can form a more compact layer. In comparison, HS-MEG-COOH has an acidic terminal group that is negatively charged in PBS; the anionic carboxyl group likely leads to a less dense layer due to repulsive forces and its bulkier size compared to the hydroxyl group. The more compact hydroxyl-terminated HS-MEG-OH SAM may enable more favourable orientations for the water molecules to interact, leading to more hydrogen bonding and reduced contact angles [28].

**Figure 3:**
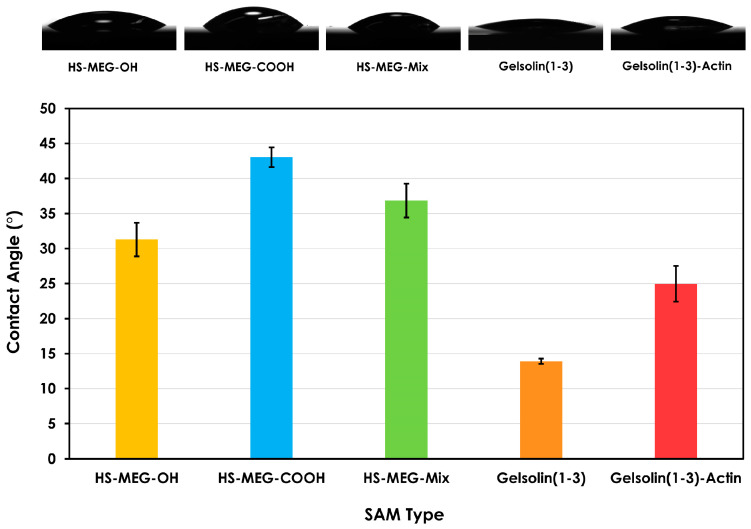
Contact angles and histogram values of SAMs on TSM-D crystals.

The wettability of the protein-functionalized surfaces is higher than that of the thiol SAMs due to the hydrophilic residues of the proteins, which enables improved interactions with water molecules. When comparing gelsolin(1-3) with gelsolin(1-3)–actin, the gelsolin-only surface is slightly more hydrophilic, with a difference of approximately 11°. The greater wettability of gelsolin(1-3) relative to gelsolin(1-3)–actin was also observed on quartz and stainless steel surfaces [16,29]. Gelsolin(1-3)–actin layers may have more hydrophobicity due to the addition of the monomeric actin protein, since globular actin has hydrophobic regions that may be exposed to the solvent [30] (Figure 3).

### 3.3. The Antifouling Properties of 3-(2-Mercaptoethanoxy)propanoic Acid (HS-MEG-COOH) with TSM-D

**Figure 4:**
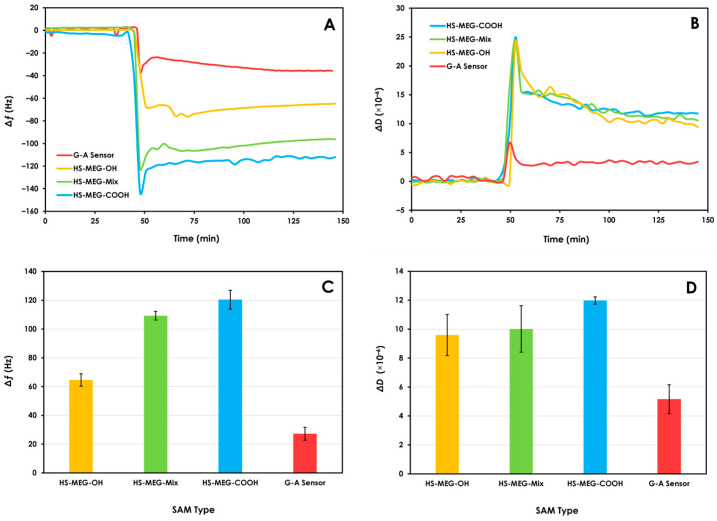
The shifts in (**A**,**C**) frequency and (**B**,**D**) dissipation upon exposing human serum to various SAM surfaces on the TSM-D crystal. The arrows in (**A**,**B**) indicate the start of the flow of undiluted human serum. The results in parts (**C**,**D**) are the mean ± SD obtained from three independent experiments in each series.

The crystal surfaces were exposed to human serum samples to study the antifouling properties of the thiols and the various surface modifications (Figure 4). Human serum was used since this biological liquid has a variety of substances, such as fats and carbohydrates, and a high protein content [31].

Considering the average frequency and dissipation variations following the fouling of human serum on the coated crystal surfaces (Table 2), HS-MEG-COOH SAMs experienced the most fouling (120.4 ± 13.0 Hz and (12.0 ± 0.5) × 10^−^^6^ frequency and dissipation shifts, respectively) compared to the HS-MEG-OH crystals (64.6 ± 8.7 Hz and (9.6 ± 2.8) × 10^−^^6^ frequency and dissipation shifts, respectively). Most likely, the negative charges of the carboxyl group participate in more interactions with some serum components and increase fouling. Previous work has shown that the substitution of a hydroxyl group with a carboxyl group caused an increase in the interaction between the surface and human serum, leading to a reduction in the antifouling properties of the layer [32]. In contrast, serum adsorption on the thiol mix (109.2 ± 6.1 Hz and (10.0 ± 3.2) × 10^−^^6^ frequency and dissipation shifts, respectively) was intermediate compared to the discs functionalized with the single thiols. These values suggest that the thiol mix was present on the surface.

There was a significant reduction in the frequency variation for serum fouling on the gelsolin(1-3)–actin protein sensor (25.3 ± 8.9 Hz and (5.2 ± 2.0) × 10^−^^6^ frequency and dissipation shifts, respectively), indicating that the protein sensor has greater antifouling behaviour. HS-MEG-COOH was linked to gelsolin(1-3)–actin via Ni-NTA, while HS-MEG-OH remained unbound; this condition of alternating longer (HS-MEG-COOH + Ni/NTA + gelsolin–actin, more precisely HS-MEG-NTA-gelsolin–actin) and shorter (HS-MEG-OH) molecules on the surface can facilitate the formation of a more extensive water barrier as water molecules may organize deeper within the layer.

**Figure 5:**
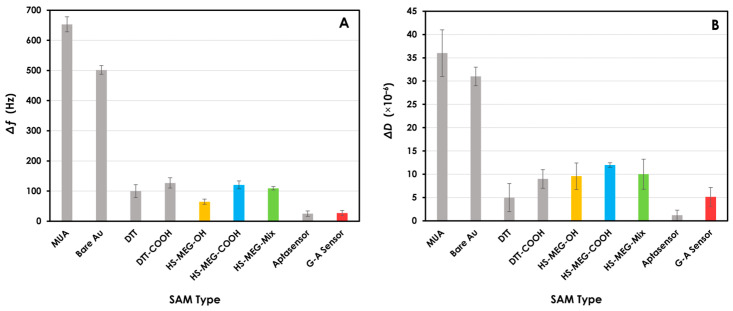
Comparing the serum fouling between the results of the present work (in colour) and the results of a previous study (grey) [21], by means of the frequency (**A**) and dissipation (**B**) changes. The G-A sensor involves the 1:1 *v*/*v* thiol mix, HS-MEG-COOH and HS-MEG-OH, in which HS-MEG-COOH was linked to the gelsolin(1-3)–actin protein complex. In the case of the aptasensor, DTT_COOH_ was linked to an aptamer. The results are the mean ± SD obtained from three independent experiments.

The variations in dissipation present the same trend as the frequency variations for the various SAM layers. A similar fouling phenomenon was observed in our recent work [33] analyzing the new antifouling thiol linker DTT_COOH_. The incorporation of the linker in a sensing layer with an aptamer bound to the carboxyl group removes the net surface charge and decreases fouling. A similar trend was also observed when HS-MEG-COOH was linked to gelsolin(1-3)–actin (the G-A sensor), and there was significantly more antifouling compared to HS-MEG-COOH, HS-MEG-OH, and HS-MEG-Mix layers (Figure 5). However, the G-A sensor used a 1:1 *v*/*v* mixture of HS-MEG-OH and HS-MEG-COOH. Since HS-MEG-COOH is linked to a large protein probe, it is expected that some HS-MEG-COOH molecules would remain unlinked. Such HS-MEG-COOH molecules with “free” terminal carboxylic acid functional groups likely become deprotonated in PBS or serum, which may explain its decreased antifouling ability compared to HS-MEG-OH due to electrostatic interactions. To reduce the number of unlinked HS-MEG-COOH linkers while also maintaining the antifouling ability of the biosensor, a mixed SAM of HS-MEG-OH and HS-MEG-COOH was applied.

In our previous work [21], bare gold surfaces showed a significant level of fouling of human serum (Figure 5). However, gold surfaces functionalized with 11-mercaptoundecanoic acid (MUA), a thiol commonly used as a linker in biosensing applications, experienced more fouling than bare gold. This highlights the importance of antifouling molecules that can also act as linkers for the application of biosensors, such as HS-MEG-COOH and DTT_COOH_.

HS-MEG-COOH and DTT_COOH_ have similar antifouling capability, with the linear molecule HS-MEG-COOH exhibiting slightly better antifouling properties. Since both molecules have carboxyl terminal groups, their similar frequency shifts from human serum adsorption were expected. In contrast, the antifouling molecule HS-MEG-OH is terminated with a hydroxyl group and experiences less serum adsorption, demonstrating how surface changes from deprotonated carboxyl groups likely participate in fouling. However, once the thiol layers are further linked to probes (aptamer for the aptasensor or gelsolin(1-3)–actin for the G-A sensor), antifouling significantly improves. The frequency shifts are relatively similar between the aptasensor and the gelsolin(1-3)–actin sensor, while the dissipation change is slightly higher for the gelsolin(1-3)–actin sensor, likely due to the different probes and thiol layers. However, both sensing surfaces demonstrate that upon the covalent immobilization of a probe, the antifouling ability of the layer considerably improves.

### 3.4. Detection of Lysophosphatidic Acid in Phosphate-Buffered Saline and Undiluted Human Serum

For the detection of the OC biomarker LPA, the gelsolin(1-3)–actin protein sensor was first tested with increasing concentrations of the biomarker in PBS and then in human serum (Table 3). The antifouling thiol layer (HS-MEG-Mix) linked to the protein complex probe enabled the detection of LPA directly in undiluted human serum. The fouling of human serum components on the sensing surface is minimal, leading to a low frequency change of ~25 Hz.

**Figure 6:**
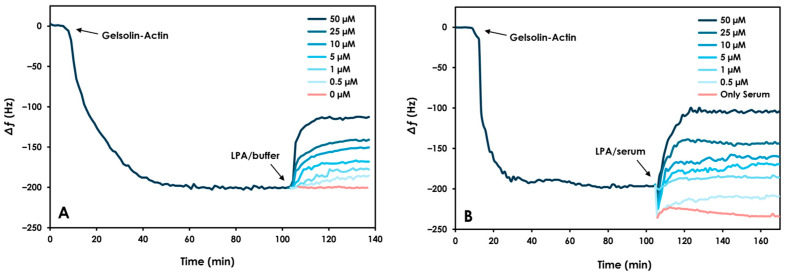
Frequency variations following the addition of (**A**) LPA-spiked PBS and (**B**) LPA-spiked human serum samples. The start of the flow of the corresponding compounds is indicated by arrows.

Since PBS does not cause fouling, LPA-spiked PBS with various LPA concentrations always increased the resonance frequency (Figure 6A). The positive frequency change is directly due to the loss of actin mass caused by the binding of LPA to the protein probe and the subsequent separation of the complex. The measurements in LPA-spiked human serum also induced frequency shifts, and the role of serum fouling is evident due to the generally lower frequency shifts in LPA-spiked serum compared to the frequency shifts in LPA-spiked PBS. Although LPA causes loss of mass on the crystal surface, slight serum fouling increases mass on the surface, which is responsible for the negative frequency change for 0.5 μM LPA in serum (Figure 6B). However, the antifouling biosensing layer significantly limits the non-specific adsorption of undiluted human serum, making quantitative detection of LPA possible.

**Figure 7:**
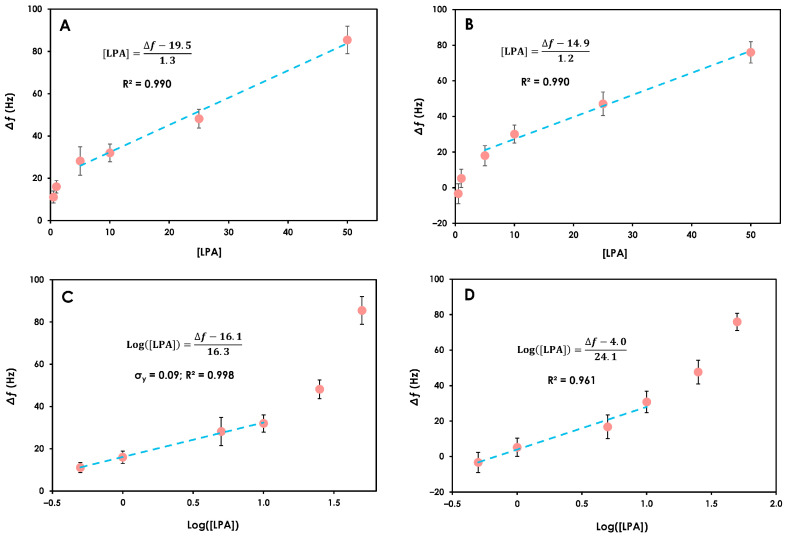
Linear calibration curves shown as dotted lines for (**A**,**C**) LPA-spiked PBS and (**B**,**D**) LPA-spiked serum measurements. (**A**,**B**) show the linear correlation between frequency shifts and higher LPA concentrations 10–50 μM, while (**C**,**D**) show the linear correlation between frequency shifts and the logarithm of LPA concentrations 0.5–10 μM. The error bars indicate the standard deviation of the measurements, which were conducted in triplicate.

Two linear models can be applied to quantify LPA in human serum samples: Figure 7B for concentrations from 10 to 50 μM LPA and Figure 7D for concentrations below 10 μM LPA. Two plots are necessary since the overall trend for frequency shifts relative to all LPA concentrations is curved. Both plots have high correlation coefficients (R^2^), indicating the ability of the sensor to successfully identify OC across all stages of the disease.

To determine the limit of detection (LOD) of the sensor, a logarithmic scale of the four lowest LPA concentrations was used since the frequency variation is large for high LPA concentrations, in particular 25 and 50 μM LPA (Figure 7C,D). The LODs in PBS and human serum were calculated from the linear regression of the plots, according to the following equation:

(1) $$\mathrm{LOD}=10^{a},\;where\;a=3.3\times \frac{\sigma }{b}$$

where *σ* is the standard deviation of the intercept on the *y*-axis, and *b* is the slope of the linear curve. Following calculations using this equation, a LOD of LPA in PBS of 1.0 μM was determined.

For measurements in human serum, a different method [34] was used to determine the LOD due to the complex medium of serum. The following equations were applied:

(2) $$\mathrm{LOB}=\mathrm{mean}\;\mathrm{blank}+1.645({\mathrm{SD}}_{\mathrm{blank}})$$

(3) $$\mathrm{LOD}=\mathrm{LOB}+1.645\;{\mathrm{SD}}_{\mathrm{lc}}$$

where the limit-of-blank (LOB) is the highest apparent concentration of LPA, SD_blank_ is the standard deviation of the blank, the mean blank is the average of the blank, and SD_lc_ is the standard deviation at the lowest concentration of the sample. To determine the concentration corresponding to the LOD, the LOD must be converted back to the concentration by applying the inverse of the regression equation and exponentiating the result:

(4) $$\mathrm{LOD}(\mathrm{in}\;\mathrm{concentration})=10^{\frac{LOD-q\;}{m}}$$

where *q* and *m* are, respectively, the intercept and the slope of the linear regression (y = *m*x + *q*).

Following Equation (2), the LOB was calculated, and this value was then used for the calculation of the LOD (Equations (3) and (4)), with a final value of approximately 0.7 μM. It is assumed that a slightly lower LOD following the detection of LPA in human serum is due to some matrix effect phenomena: LPA may bind better to the protein complex and dissociate it in a more natural environment like human serum. It may also be that serum affects the stability of the complex and makes it more sensitive to disassembly. However, these phenomena need to be investigated and can be the subject of future studies. The detected LOD is certainly below the limit of detection for distinguishing healthy LPA levels (~1.2 μM) from the presence of OC, since the disease can have upregulated LPA concentrations of 1.3 to 50 μM [17,35,36].

## 4. Conclusions

A thickness shear mode acoustic sensor with dissipation monitoring (TSM-D) was used to develop an assay for detecting the early-stage ovarian cancer biomarker, lysophosphatidic acid (LPA). The protein complex gelsolin–actin enabled the indirect quantitative detection of LPA since LPA causes separation of the complex, leading to loss of actin protein mass on the sensing surface. LPA was detected in phosphate-buffered saline and undiluted human serum with similar LODs of 1.0 and 0.7 μM, respectively. The antifouling thiol molecules HS-MEG-COOH and HS-MEG-OH were incorporated in the gelsolin–actin sensor, where HS-MEG-COOH enabled the covalent immobilization of the gelsolin–actin probe via its carboxyl terminal group. HS-MEG-COOH is a new antifouling thiol molecule that demonstrated excellent antifouling properties in undiluted human serum, which was significantly improved once the molecule was linked to the protein probe. The antifouling and linking capability of HS-MEG-COOH are important characteristics for biosensors with gold sensing surfaces, such as TSM or gold screen-printed electrodes. The successful application of the new linker molecule in this work is demonstrated by the ability to directly detect LPA in undiluted human serum, in which the low limit of detection of the developed biosensor enables the identification of nascent ovarian cancer.

## Figures and Tables

**Table 1:** Values of contact angle of SAMs on TSM-D crystals at various modifications. Results are the mean ± SD obtained from three independent experiments.

Surface	Angle (^°^)	RSD (%)
HS-MEG-OH	31.3 ± 4.8	15.3
HS-MEG-COOH	43.0 ± 2.8	6.5
HS-MEG-Mix	36.8 ± 4.8	13.0
Gelsolin(1-3)	13.9 ± 0.7	5.0
Gelsolin(1-3)–Actin	25.0 ± 5.1	20.4

**Table 2:** The changes in frequency, Δ*f*, and dissipation, Δ*D*, after exposing various self-assembled monolayers to undiluted human serum. The results are the mean ± SD obtained from three independent experiments.

SAM	Δ*f* (Hz)	RSD (%)	Δ*D* (10^−6^)	RSD (%)
HS-MEG-COOH	120.4 ± 13.0	10.8	12.0 ± 0.5	4.2
HS-MEG-OH	64.6 ± 8.7	13.5	9.6 ± 2.8	29.2
HS-MEG-Mix	109.2 ± 6.1	5.6	10.0 ± 3.2	32.0
Gelsolin(1-3)–actin	25.3 ± 8.9	35.2	5.2 ± 2.0	38.5

**Table 3:** The changes in frequency, Δ*f*, after exposing the gelsolin(1-3)–actin protein sensor to various concentrations of LPA in PBS or undiluted human serum. The results are the mean ± SD obtained from three independent experiments.

LPA (µM)	∆*f* in PBS (Hz)	RSD (%)	∆*f* in Serum (Hz)	RSD (%)
0 (Only serum)	0.0 ± 0.5	N/A *	−25.3 ± 8.9	35.2
0.5	11.1 ± 1.2	10.8	−3.4 ± 5.6	164.7
1	16.0 ± 1.5	9.4	5.2 ± 5.1	98.1
5	28.2 ± 6.7	23.8	16.8 ± 6.6	39.3
10	32.0 ± 2.1	6.6	30.8 ± 6.1	19.8
25	48.2 ± 4.4	9.1	47.6 ± 6.6	13.9
50	85.4 ± 6.5	7.6	75.9 ± 4.8	6.3

* The relative standard deviation is not applicable to serum without LPA since there is no variation in frequency.

## Data Availability

Data are contained within this article.

## Supplementary Materials

The following supporting information can be downloaded at https://www.mdpi.com/article/10.3390/s24247884/s1. S1: The NMR spectra of the compounds used for the synthesis of the antifouling linker HS-MEG-COOH. ^1^H and ^13^C NMR spectra were recorded at room temperature using either a Bruker Avance III 400 MHz Spectrometer or 500 MHz Agilent DD2 Spectrometer using CDCl_3_ with TMS as the NMR solvent. ^1^H and ^13^C NMR spectra are referenced to the residual solvent peaks (CDCl_3_: 7.26 and 77.16 ppm, respectively). Figure S1.1. ^1^H NMR spectrum (CDCl_3_ with TMS, 500 MHz) for 2,2′-disulfanediylbis(ethan-1-ol) (DS-2EtOH): δ 3.90 (t, 4H), 2.88 (t, 4H), 2.39 (s, 2H) ppm. Figure S1.2. ^13^C NMR spectrum (CDCl_3_ with TMS, 125 MHz) for 2,2′-disulfanediylbis(ethan-1-ol) (DS-2EtOH): δ 60.5, 41.4 ppm. Figure S1.3. ^1^H NMR spectrum (CDCl_3_ with TMS, 500 MHz) for di-tert-butyl-3,3′-((disulfanediylbis(ethane-2,1-diyl))bis(oxy)) dipropionate (DS-2EtOEtCOOTBu): δ 3.72-3.65(m, 8H), 2.85 (t, 4H), 2.48 (t, 4H), 1.44 (s, 18H) ppm. Figure S1.4. ^13^C NMR spectrum (CDCl_3_ with TMS, 125 MHz) for di-tert-butyl-3,3′-((disulfanediylbis(ethane-2,1-diyl))bis(oxy)) dipropionate (DS-2EtOEtCOOTBu): δ 170.9, 80.7, 69.4, 66.8, 38.6, 36.4, 28.2 ppm. Figure S1.5. ^1^H NMR spectrum (CDCl_3_ with TMS, 500 MHz) for 3,3′-((disulfanediylbis(ethane-2,1-diyl))bis(oxy))dipropionic acid (DS-2EtOEtCOOH): δ 3.79 (t, 4H), 3.73 (t, 4H), 2.88 (t, 4H), 2.65 (t, 4H) ppm. Figure S1.6. ^13^C NMR spectrum (CDCl_3_ with TMS, 125 MHz) for 3,3′-((disulfanediylbis(ethane-2,1-diyl))bis(oxy))dipropionic acid (DS-2EtOEtCOOH): δ 177.4, 69.5, 66.3, 38.7, 35.1 ppm. Figure S1.7. ^1^H NMR spectrum (CDCl_3_ with TMS, 400 MHz) for 3-(2-mercaptoethanoxy)propanoic acid (HS-MEG-COOH): δ 3.74 (t, 2H), 3.60 (t, 2H), 2.71-2.59 (m, 4H), 1.57 (s, 1H) ppm. Figure S1.8. ^13^C NMR spectrum (CDCl_3_ with TMS, 100 MHz) for 3-(2-mercaptoethanoxy)propanoic acid (HS-MEG-COOH): δ 176.4, 72.8, 66.0, 34.9, 24.3 ppm. S2: The FTIR analysis of HS-MEG-COOH. HS-MEG-COOH was characterized by Fourier-transform infrared spectroscopy (Prestige-21 FTIR spectrometer from Shimadzu, Kyoto, Japan). HS-MEG-COOH was dissolved in ethanol (4 mM concentration) and poured dropwise onto the CaF_2_ cell (Specac, Orpington, UK). The transmittance mode measurement was conducted following the evaporation of the solvent. Figure S2.1 shows the FTIR spectrum, where the functional groups of HS-MEG-COOH are labeled. Figure S2.1. FTIR transmittance spectrum of HS-MEG-COOH following evaporation of ethanol. S3: The antifouling analysis of self-assembled monolayers (SAMs). While wettability does not indicate the antifouling ability of a surface, a plot comparing the contact angles and frequency shifts after incubation with human serum may provide some correlation. However, contact angle goniometry is more appropriate for understanding whether functionalization with a different monolayer occurred. Figure S3.1. Comparing the frequency shifts following human serum fouling with the contact angles of various SAMs. The antifouling behaviour of the HS-MEG-COOH and HS-MEG-OH mixtures was compared to determine the most optimal ratio. The fouling of human serum was similar for the 1:1, 1:2, and 1:3 mixtures of HS-MEG-COOH:HS-MEG-OH. The 2:1 mixture experienced the most fouling. The 1:1 ratio was used in the experiments to have more HS-MEG-COOH linkers available for binding the protein probe while also maintaining the antifouling properties of the layer. Figure S3.2. The frequency shifts following human serum for different ratios of HS-MEG-COOH and HS-MEG-OH SAMs. Figure S3.3. The frequency shifts for different mixtures of HS-MEG-COOH and HS-MEG-OH SAMs due to the fouling of human serum.
