# Supplementary material for: Acoustic Wave Sensor Detection of an Ovarian Cancer Biomarker with Antifouling Surface Chemistry
Source: Sensors (Basel). 2024 Dec 10;24(24):7884. doi: 10.3390/s24247884 (PMC11678973; doi:10.3390/s24247884)
Supplement: Supplementary file 1 [file sensors-24-07884-s001.zip › sensors-3304728-supplementary.pdf]

Supplementary Material

Acoustic Wave Sensor Detection of an Ovarian Cancer Biomarker with Antifouling Surface Chemistry

Katharina Davoudian [1], Sandro Spagnolo [2], Edmund Chan [1], Tibor Hianik [2] and Michael Thompson [1,*]

[1] Department of Chemistry, University of Toronto, 80 St. George Street, Toronto, ON M5S 3H6, Canada; k.davoudian@mail.utoronto.ca (K.D.); edmund.chan@mail.utoronto.ca (E.C.)
[2] Faculty of Mathematics, Physics and Informatics, Comenius University, Mlynská dolina F1,
842 48 Bratislava, Slovakia; spagnolo2@uniba.sk (S.S.); tibor.hianik@fmph.uniba.sk (T.H.)
* Correspondence: m.thompson@utoronto.ca

S1. NMR spectra of the compounds used for the synthesis of the antifouling linker HS-MEG-COOH

^{1}H and ^{13}C NMR spectra were recorded at room temperature using either Bruker Avance III 400 MHz Spectrometer or 500 MHz Agilent DD2 Spectrometer using $CDCl_3$ with TMS) as the NMR solvent. ^{1}H and ^{13}C NMR spectra are referenced to the residual solvent peaks ($CDCl_3$: 7.26 and 77.16 ppm, respectively).

2,2'-disulfanediylbis(ethan-1-ol) (DS-2EtOH): ^{1}H NMR ($CDCl_3$ with TMS, 500 MHz): δ 3.90 (t, 4H), 2.88 (t, 4H), 2.39 (s, 2H) ppm**.** ^{13}C NMR ($CDCl_3$ with TMS, 125 MHz): δ 60.5, 41.4 ppm.

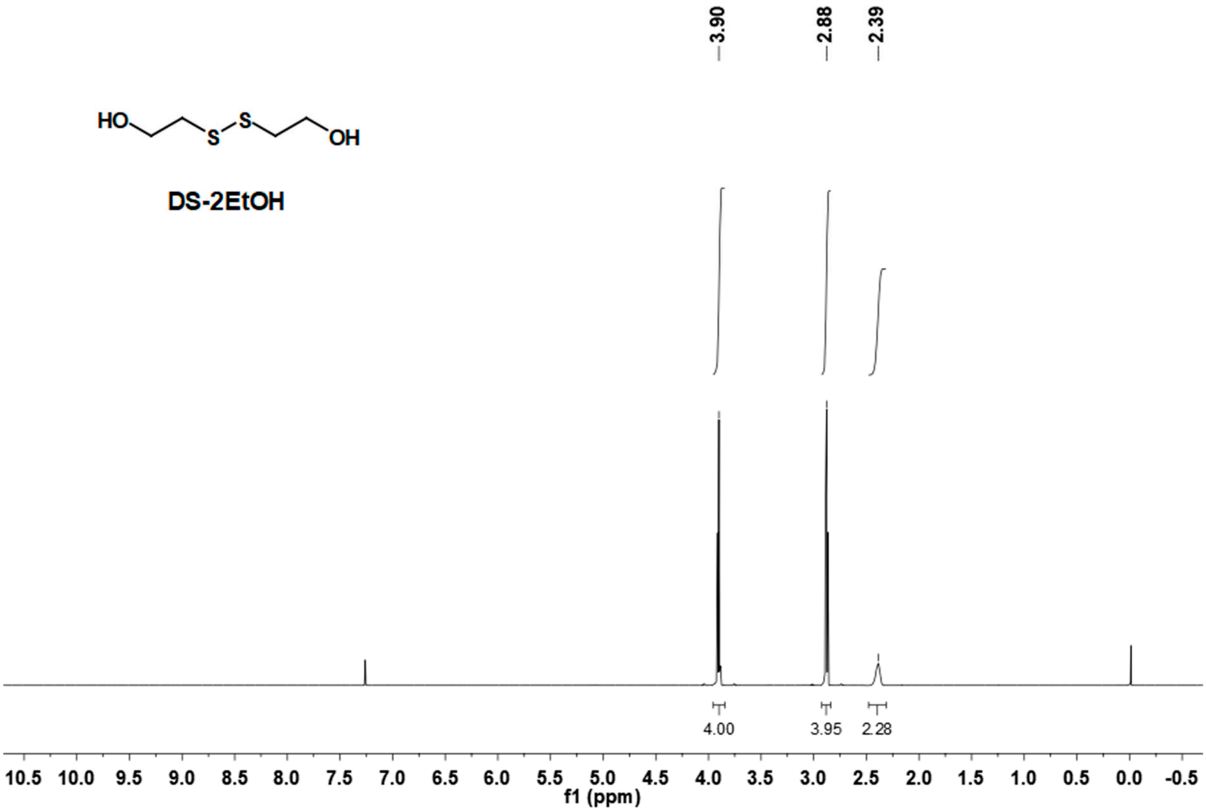

Figure S1.1. ^{1}H NMR spectrum for DS-2EtOH.

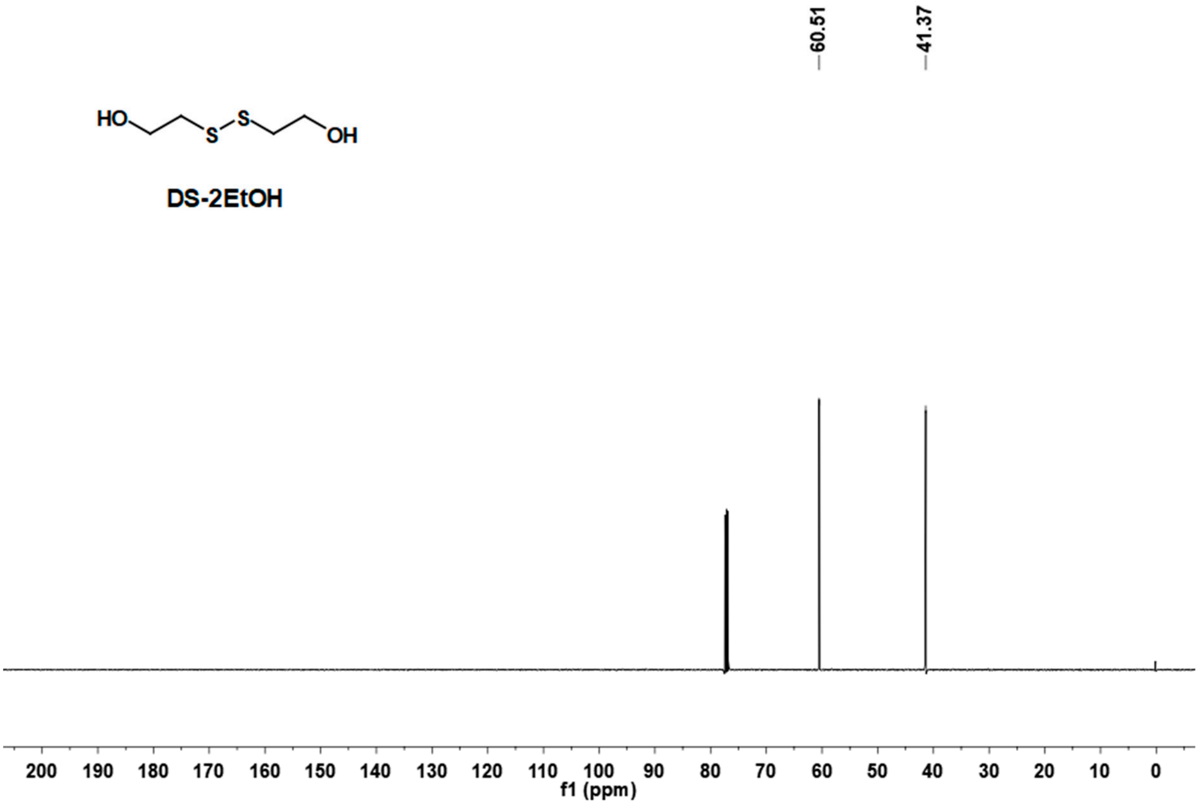

Figure S1.2. ^{13}C NMR spectrum for DS-2EtOH.

Di-tert-butyl-3,3'-((disulfanediylbis(ethane-2,1-diyl))bis(oxy)) dipropionate (DS-2EtOEtCOOTBu): ^{1}H NMR ($CDCl_3$ with TMS, 500 MHz): δ 3.72-3.65(m, 8H), 2.85 (t,

4H), 2.48 (t, 4H), 1.44 (s, 18H) ppm. ^{13}C NMR ($CDCl_3$ with TMS, 125 MHz): δ 170.9, 80.7, 69.4, 66.8, 38.6, 36.4, 28.2 ppm.

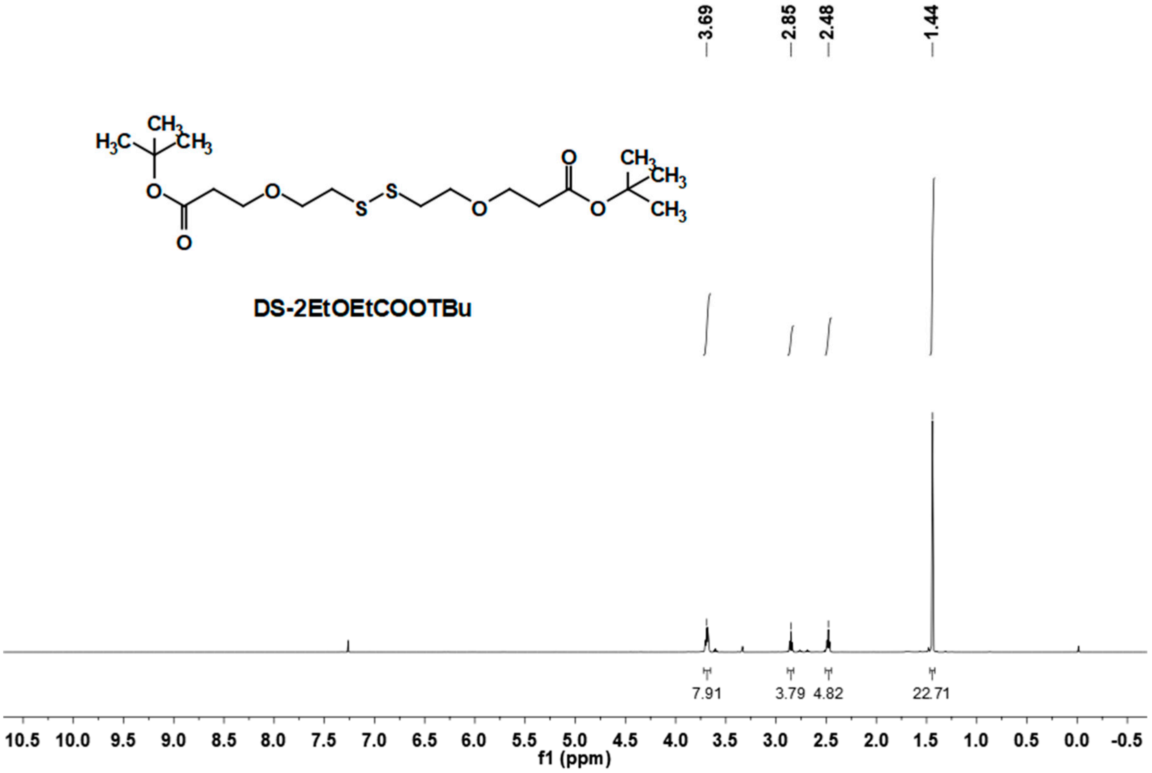

Figure S1.3. ^{1}H NMR spectrum for DS-2EtOEtCOOTBu.

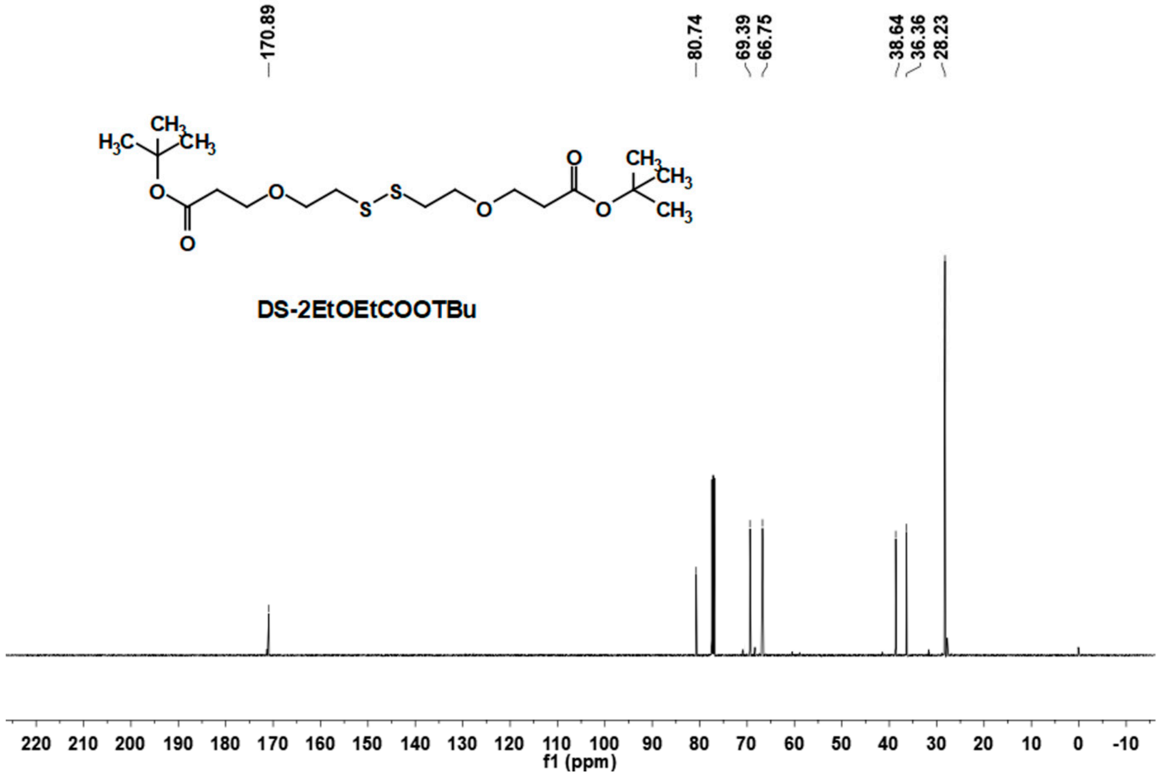

Figure S1.4. ^{13}C NMR spectrum for DS-2EtOEtCOOTBu.

3,3'-((disulfanediylbis(ethane-2,1-diyl))bis(oxy))dipropionic acid (DS-2EtOEtCOOH): ^{1}H NMR ($CDCl_3$ with TMS, 500 MHz): δ 3.79 (t, 4H), 3.73 (t, 4H), 2.88 (t, 4H), 2.65 (t, 4H) ppm. ^{13}C NMR ($CDCl_3$ with TMS, 125 MHz): δ 177.4, 69.5, 66.3, 38.7, 35.1 ppm.

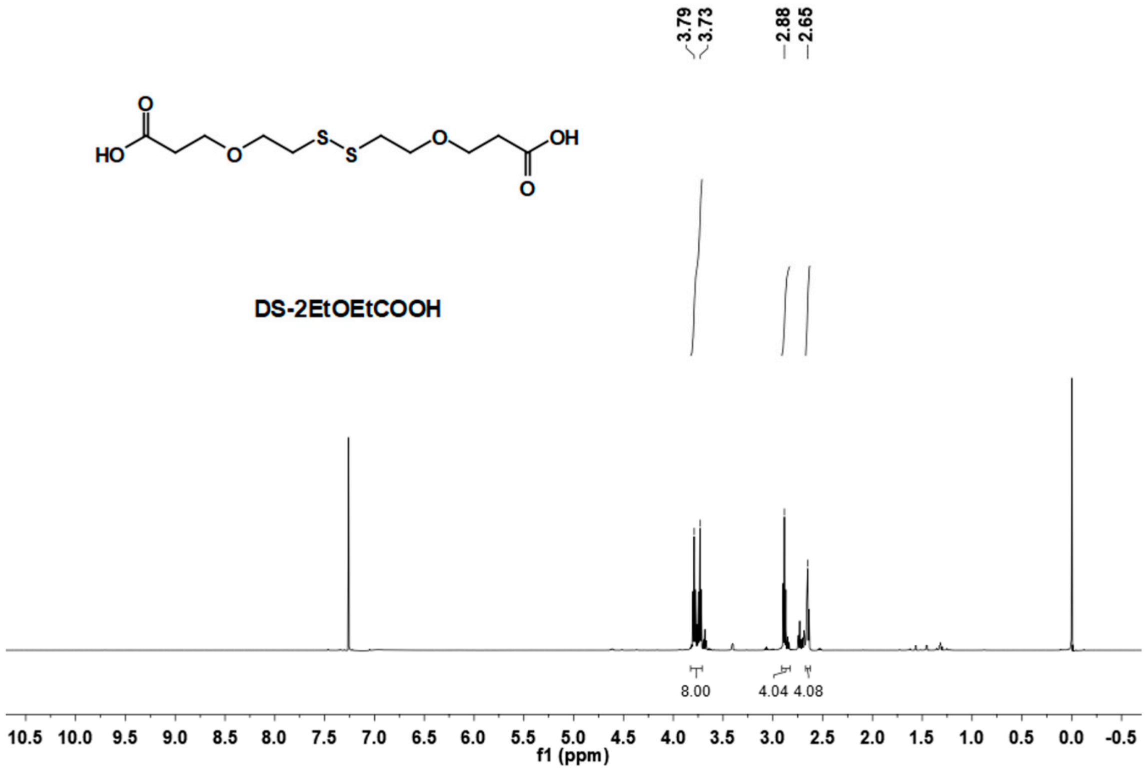

Figure S1.5. ^{1}H NMR spectrum for DS-2EtOEtCOOH.

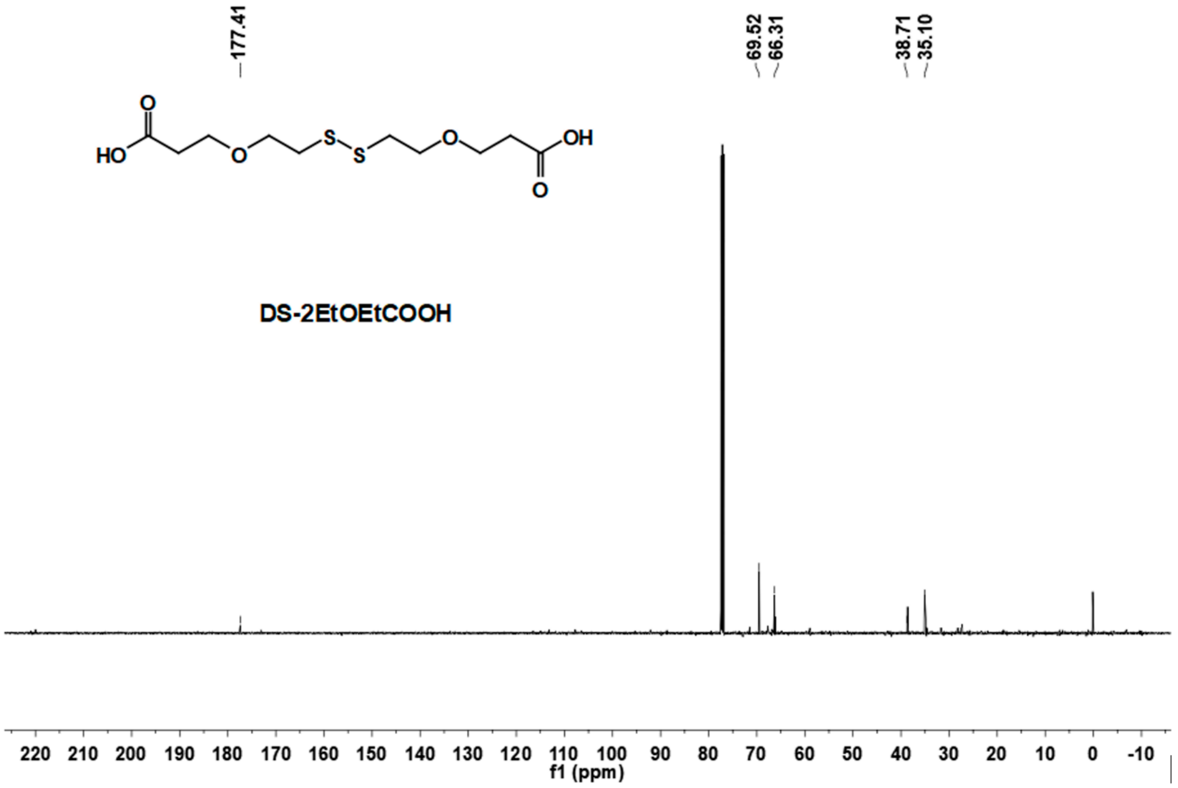

Figure S1.6. ^{13}C NMR spectrum for DS-2EtOEtCOOH.

3-(2-mercaptoethanoxy)propanoic acid (HS-MEG-COOH): ^{1}H NMR ($CDCl_3$ with TMS, 400 MHz): δ 3.74 (t, 2H), 3.60 (t, 2H), 2.71-2.59 (m, 4H), 1.57 (s, 1H) ppm. ^{13}C NMR ($CDCl_3$ with TMS, 100 MHz): δ 176.4, 72.8, 66.0, 34.9, 24.3 ppm.

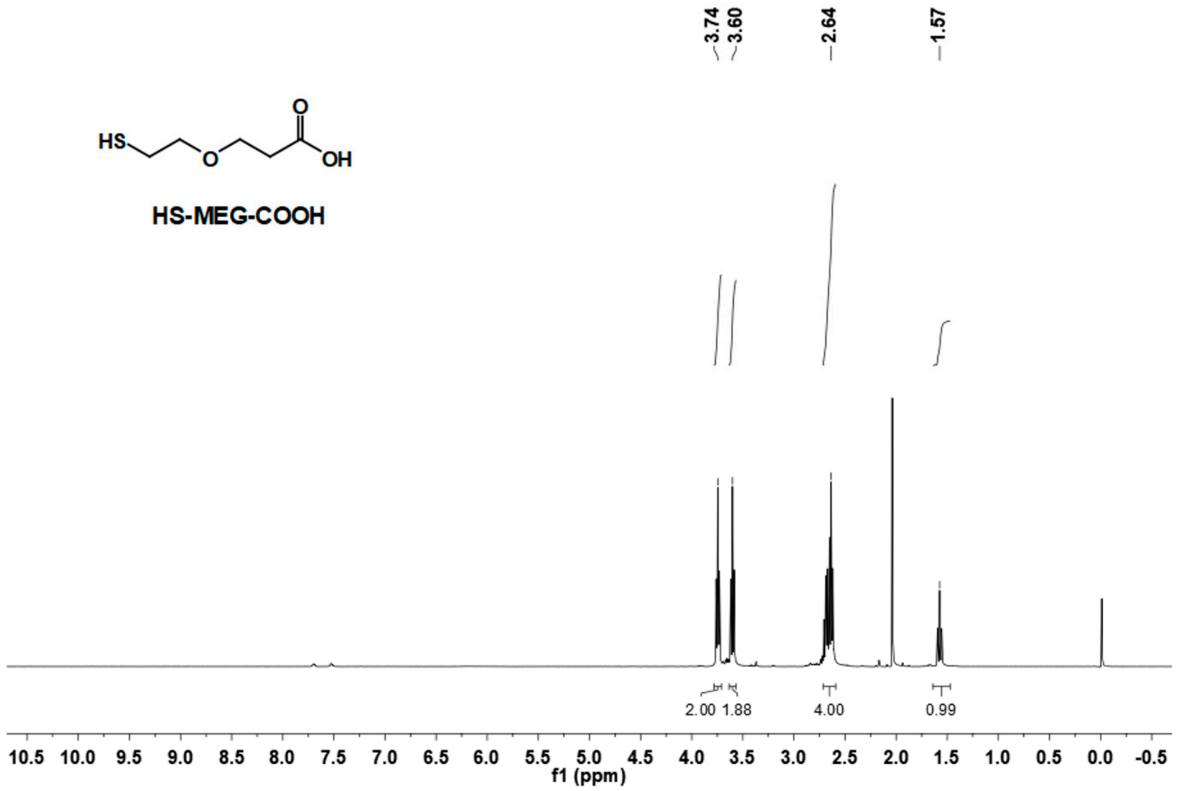

Figure S1.7. ^{1}H NMR spectrum for HS-MEG-COOH.

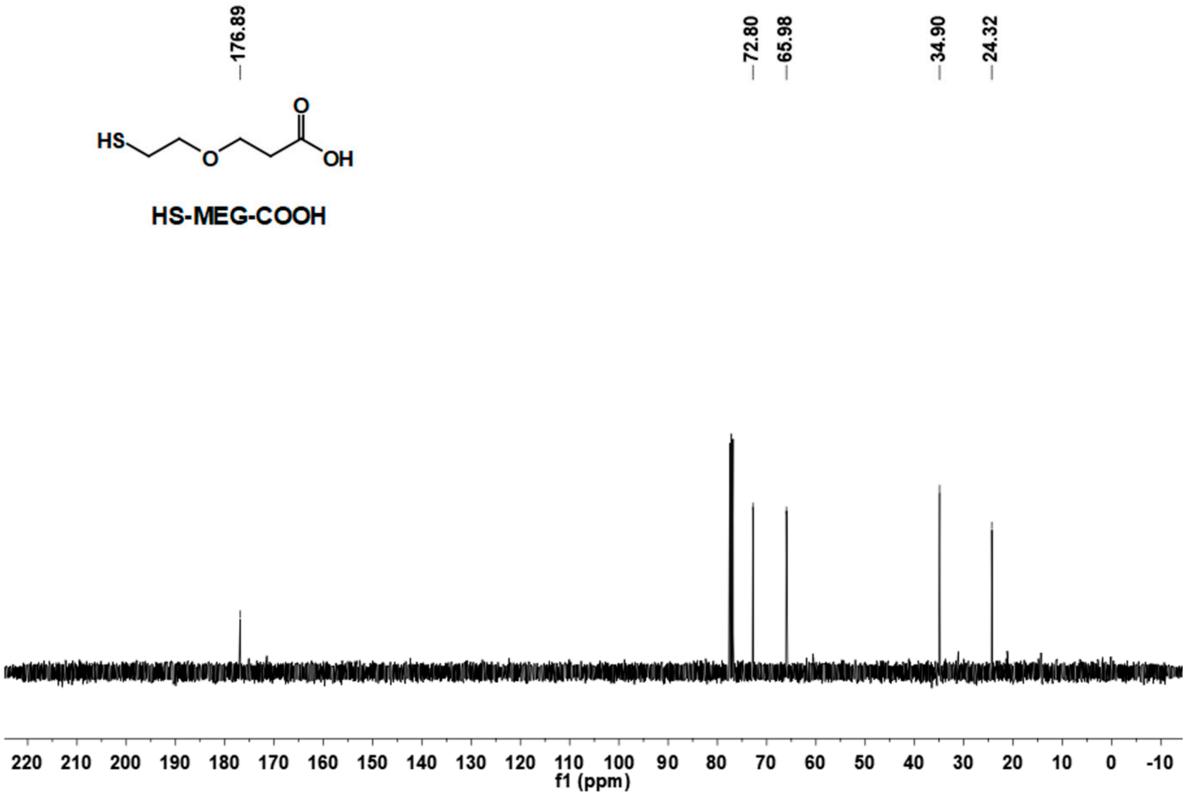

Figure S1.8. ^{13}C NMR spectrum for HS-MEG-COOH.

S2. FTIR Analysis of HS-MEG-COOH

HS-MEG-COOH was characterized by Fourier-transform infrared spectroscopy (Prestige-21 FTIR spectrometer from Shimadzu, Kyoto, Japan). HS-MEG-COOH was dissolved in ethanol (4 mM concentration) and poured dropwise onto a CaF_2 cell (Specac, Orpington, UK). The transmittance mode measurement was done following evaporation of the solvent. **Figure S2.1** shows the FTIR spectrum, where the functional groups of HS-MEG-COOH are labeled.

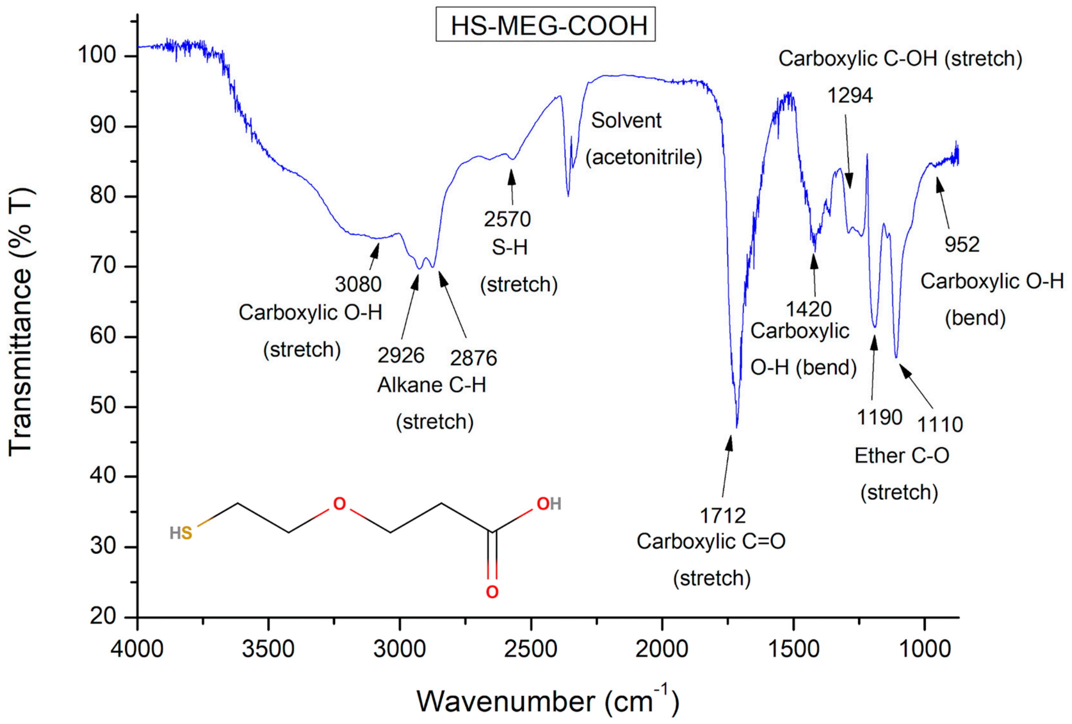

Figure S2.1. FTIR transmittance spectrum of HS-MEG-COOH following evaporation of ethanol.

S3. Antifouling Analysis of Self-Assembled Monolayers (SAMs)

While wettability does not indicate the antifouling ability of a surface, a plot comparing the contact angles and frequency shifts after incubation with human serum may provide some correlation. However, contact angle goniometry is more appropriate for understanding whether functionalization with a different monolayer occurred.

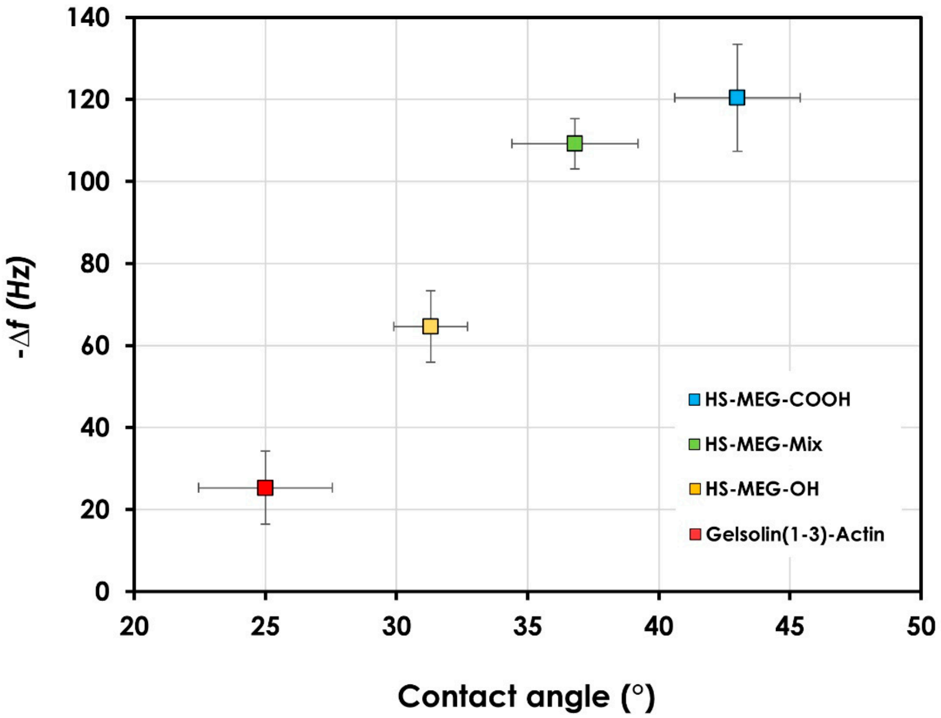

Figure S3.1. Comparing the frequency shifts following human serum fouling with the contact angles of various SAMs.

The antifouling behaviour of HS-MEG-COOH and HS-MEG-OH mixtures was compared to determine the most optimal ratio. Fouling of human serum was similar for 1:1, 1:2, and 1:3 mixtures of HS-MEG-COOH:HS-MEG-OH. The 2:1 mixture experienced the most fouling. The 1:1 ratio was used in the experiments to have more HS-MEG-COOH linkers available for binding the protein probe while also maintaining the antifouling properties of the layer.

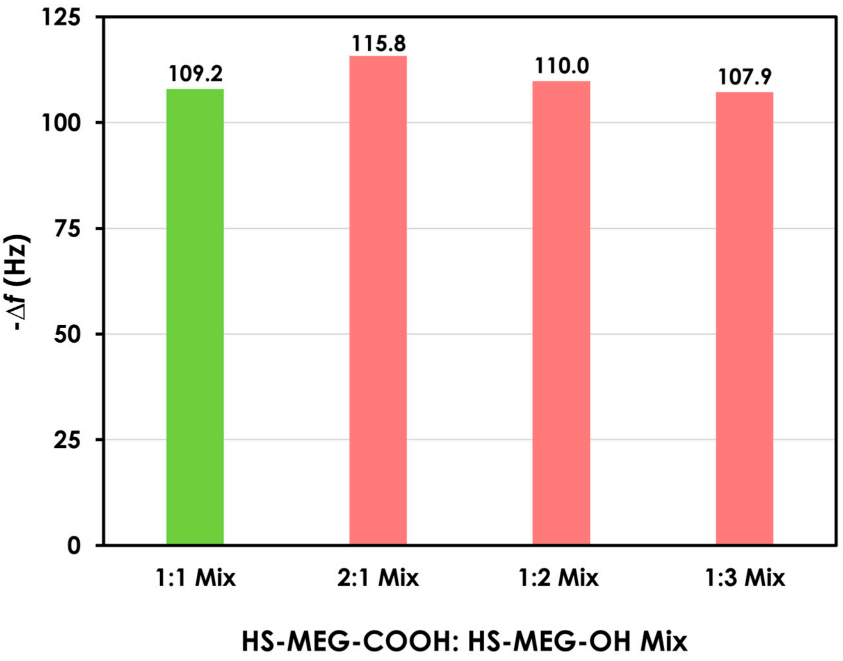

Figure S3.2. The frequency shifts following undiluted human serum for different ratios of HS-MEG-COOH and HS-MEG-OH SAMs.

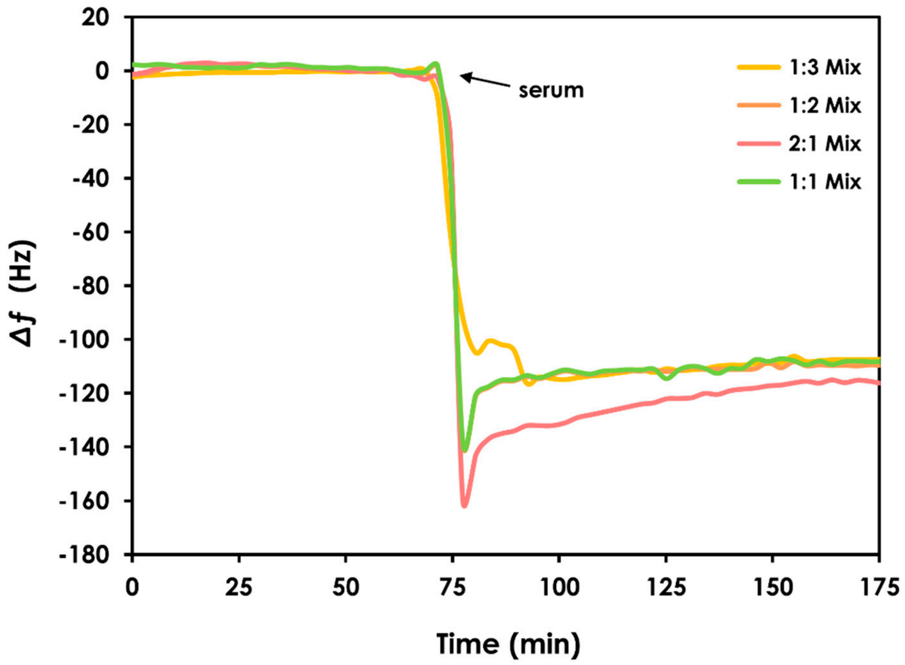

Figure S3.3. The frequency shifts for different mixtures of HS-MEG-COOH and HS-MEG-OH SAMs due to the fouling of undiluted human serum. The start of the flow of the human serum is indicated by an arrow.
